# 1D Copper(II)-Aroylhydrazone Coordination Polymers: Magnetic Properties and Microwave Assisted Oxidation of a Secondary Alcohol

**DOI:** 10.3389/fchem.2020.00157

**Published:** 2020-03-06

**Authors:** Manas Sutradhar, Elisabete C. B. A. Alegria, Tannistha Roy Barman, M. Fátima C. Guedes da Silva, Cai-Ming Liu, Armando J. L. Pombeiro

**Affiliations:** ^1^Centro de Química Estrutural, Instituto Superior Técnico, Universidade de Lisboa, Lisbon, Portugal; ^2^Área Departamental de Engenharia Química, Instituto Superior de Engenharia de Lisboa, Instituto Politécnico de Lisboa, Lisbon, Portugal; ^3^National Laboratory for Molecular Sciences, Center for Molecular Science, Institute of Chemistry, Chinese Academy of Sciences, Beijing, China

**Keywords:** Cu(II) complexes, coordination polymer, X-ray structure, magnetism, microwave assisted oxidation of alcohols

## Abstract

The 1D Cu(II) coordination polymers [Cu_3_(L^1^)(NO_3_)_4_(H_2_O)_2_]_n_ (**1**) and [Cu_2_(H_2_L^2^)(NO_3_)(H_2_O)_2_]_n_(NO_3_)_n_ (**2**) have been synthesized using the aroylhyrazone Schiff bases *N'*^1^,*N'*^2^-bis(pyridin-2-ylmethylene)oxalohydrazide (H_2_L^1^) and *N'*^1^,*N'*^3^-bis(2-hydroxybenzylidene)malonohydrazide (H_4_L^2^), respectively. They have been characterized by elemental analysis, infrared (IR) spectroscopy, UV-Vis spectroscopy, electrospray ionization mass spectrometry (ESI-MS), single crystal X-ray diffraction and variable temperature magnetic susceptibility measurements (for **2**). The ligand (L^1^)^2−^ coordinates in the *iminol* form in **1**, whereas the *amide* coordination is observed for (H_2_L^2^)^2−^ in **2**. Either the ligand bridge or the nitrate bridge in **2** mediates weak antiferromagnetic coupling. The catalytic performance of **1** and **2** has been investigated toward the solvent-free microwave-assisted oxidation of a secondary alcohol (1-phenylethanol used as model substrate). At 120°C and in the presence of the nitroxyl radical 2,2,6,6-tetramethylpiperydil-1-oxyl (TEMPO), the complete conversion of 1-phenylethanol into acetophenone occurs with TOFs up to 1,200 h^−1^.

**Graphical Abstract:**
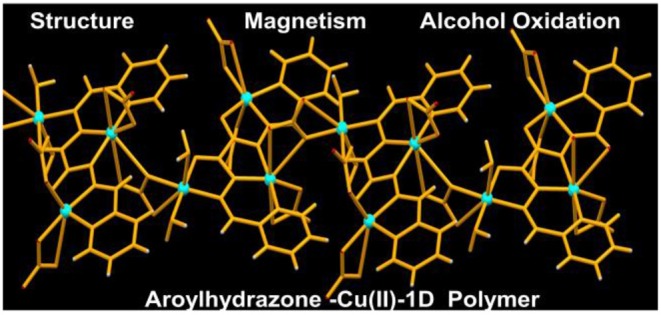
Two new aroylhydrazone Cu(II) 1D coordination polymers [Cu_3_(L^1^)(NO_3_)_4_(H_2_O)_2_]_n_ (**1**) and [Cu_2_(H_2_L^2^)(NO_3_)(H_2_O)_2_]_n_·(NO_3_)_n_ (**2**) (H_2_L^1^ = *N'*^1^,*N'*^2^-bis(pyridin-2-ylmethylene)oxalohydrazide and H_4_L^2^ = *N'*^1^,*N'*^3^-bis(2-hydroxybenzylidene)malonohydrazide) have been structurally characterized. Their magnetic properties and the catalytic activity toward the solvent-free microwave-assisted oxidation of 1-phenylethanol have been explored.

## Introduction

Transition metal complexes derived from multidentate Schiff base ligands received high significance due to their wide dimension of applications in the areas of molecular magnetism (Benelli and Gatteschi, 2002; Sutradhar et al., 2012, 2013, 2014b, 2015a, 2018; Cho et al., 2016; Andruh, 2018), crystal engineering (Dong et al., 2000; Kitaura et al., 2004; Andruh et al., 2009), supramolecular chemistry (Pradeep and Das, 2013; El-Bindary et al., 2016; Dwivedi et al., 2018), catalysis (Sutradhar et al., 2013, 2014b, 2015a,b,c, 2016a,b,c, 2017, 2018, 2019), etc. Molecular magnetism is one of the significant domains determining magneto-structural correlations to design magnetic materials (Benelli and Gatteschi, 2002; Sutradhar et al., 2012, 2013, 2014b, 2015a, 2018; Cho et al., 2016; Andruh, 2018). Single molecule magnets (SMMs) (Karotsis et al., 2010; Glaser et al., 2015; Maniaki et al., 2018) exhibit a large spin ground-state (*S*) value and large negative magnetic anisotropy and are used in potential high-density information storage devices and quantum computers (Leuenberger and Loss, 2001). Therefore, many efforts have been devoted to rationally establish polynuclear metal complexes and/or clusters elucidating molecular magnetism (Benelli and Gatteschi, 2002; Karotsis et al., 2010; Sutradhar et al., 2012, 2013, 2014b, 2015a, 2018; Glaser et al., 2015; Cho et al., 2016; Andruh, 2018; Maniaki et al., 2018). Coordination polymers and molecular clusters are particular classes of compounds that can display novel magnetic properties including high ground-state spin values and single molecule magnetism behavior (Zheng et al., 2014; Journaux et al., 2018; Yue and Gao, 2019).

The coordination behavior of Cu(II) is versatile, flexible and magnetically attractive. Many Cu(II) complexes are widely explored for magnetic studies focused on the synthesis of polynuclear complexes and/or clusters and their possible application in molecular magnetism (Benelli and Gatteschi, 2002; Sutradhar et al., 2013, 2015a, 2018; Andruh, 2018). Several attempts have been carried out to synthesize polynuclear Cu(II) complexes by designing suitable flexidentate Schiff base ligands to fabricate magnetic properties (Lu et al., 2013; Sutradhar et al., 2013, 2015a, 2018). Moreover, some multinuclear copper complexes show good catalytic performances toward the mild peroxidative oxidation of alkanes and alcohols into more valuable organic products (Pombeiro, 2013, 2019; Sutradhar et al., 2013, 2015a, 2018; Kopylovich et al., 2015) thus contributing to one of the most challenging subjects of modern chemistry. However, such examples are still limited and the study requires further exploration.

Aroylhydrazone Schiff bases are versatile in terms of functionalities, coordination properties and chelating abilities (Sutradhar et al., 2013, 2015a,b,c, 2016a,b,c, 2017, 2018). In continuation of our work in the fields of catalysis and magnetic studies, herein we report the syntheses of two 1D coordination polymers using two different multidentate *N*,*O* donor aroylhydrazone Schiff bases. The magnetic properties of one of them (complex **2**) are studied using variable temperature magnetic susceptibility measurements. These 1D polymers are also tested as catalysts toward the microwave assisted peroxidative oxidation of alcohols under mild conditions for the future development of an environment benign catalytic system.

## Experimental

All synthetic work was performed in air. Commercially available reagents and solvents were used as received, without further purification or drying. Cu(NO_3_)_2_·2.5H_2_O was used as metal source for the synthesis of the complexes.

C, H, and N elemental analyses were carried out by the Microanalytical Service of Instituto Superior Técnico. Infrared spectra (4,000–400 cm^−1^) were recorded on a Bruker Vertex 70 (Bruker Corporation, Ettlingen, Germany) instrument in KBr pellets; wavenumbers are in cm^−1^. The ^1^H NMR spectra were recorded at room temperature on a Bruker Avance II + 400.13 MHz (UltraShieldTM Magnet, Rheinstetten, Germany) spectrometer. The UV–Vis absorption spectra of methanol solutions of **1** and **2** (*ca*. 2 × 10^−5^M) in 1.00 cm quartz cells were recorded at room temperature on a Lambda 35 UV–Vis spectrophotometer (Perkin–Elmer) by scanning the 200–1000 nm region at a rate of 240 nm min^−1^. Tetramethylsilane was used as the internal reference and the chemical shifts are reported in ppm. Mass spectra were run in a Varian 500-MS LC Ion Trap Mass Spectrometer (Agilent Technologies, Amstelveen, The Netherlands) equipped with an electrospray (ESI) ion source. For electrospray ionization, the drying gas and flow rate were optimized according to the particular sample with 35 p.s.i. nebulizer pressure. Scanning was performed from *m/z* 100 to 1,200 in methanol solution. The compounds were observed in the positive mode (capillary voltage = 80–105 V). The catalytic tests were performed under microwave (MW) irradiation using a focused Anton Paar Monowave 300 microwave (Anton Paar GmbH, Graz, Austria) fitted with a rotational system and an IR temperature detector, using a 10 mL capacity reaction tube with a 13 mm internal diameter. Gas chromatographic (GC) measurements were carried in a FISONS Instrument GC 8000 series gas chromatograph with a capillary DB-WAX column (30 m x 0.32 mm), a FID detector, helium as the carrier gas and using the Jasco-Borwin v.1.50 software. The magnetic susceptibility measurements were carried out on a polycrystalline samples with a Quantum Design MPMS-XL5 SQUID magnetometer in the temperature range of 2–300 K and at an applied field of 2000 Oe. Diamagnetic corrections were estimated from Pascal's constants for all constituent atoms (Kahn, 1993).

### Synthesis of the *N'*^1^,*N'*^2^-bis(pyridin-2-ylmethylene)oxalohydrazide (H_2_L^1^)

**Scheme 1:**
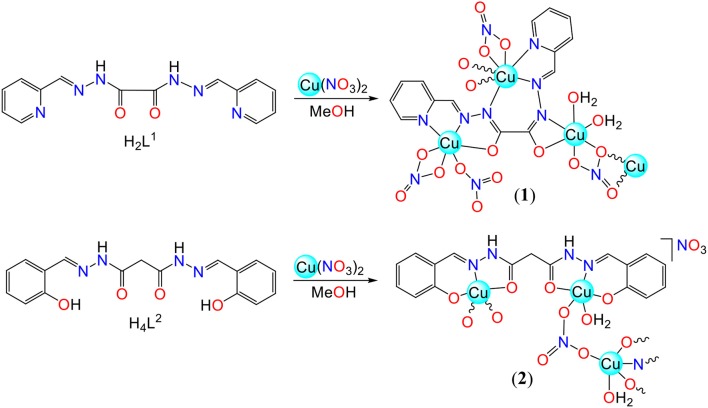
Syntheses of **1** and **2**.

The aroylhydrazone Schiff base pro-ligand H_2_L^1^ (Scheme 1) was prepared by using a similar method reported for the synthesis of *N'*^1^,*N'*^3^-bis(2-hydroxybenzylidene)malonohydrazide (H_4_L^2^) (Sutradhar et al., 2014a) upon condensation of the oxalyl dihydrazide with pyridine-2-aldehyde.

Yield: 86%. Anal. Calcd for H_2_L^1^ C_14_H_12_N_6_O_2_: C, 56.75; H, 4.08; N, 28.36. Found: C, 56.68; H, 4.04; N, 28.29. IR (KBr pellet, cm^−1^): 3131ν(NH), 1681 ν(C=O) 1185 ν(N–N). ^1^H NMR (DMSO-d6, δ): 12.67 (s, 2H, NH), 7.47 (s, 2H, -CH=N), 8.67–7.89 (m, 8H, C_5_H_4_N). UV–Vis _max_ (MeOH, nm (ε, LM^−1^ cm^−1^)): 656 (305), 378 (16,478), 285 (23,434), 237 (32,108). ESI-MS(+): *m/z* 297 [M+H]^+^ (100%).

### Synthesis of [Cu_3_(L^1^)(NO_3_)_4_(H_2_O)_2_]_n_ (1)

0.930 g Cu(NO_3_)_2_·2.5H_2_O (4.0 mmol) was added to a 25 mL methanolic suspension of H_2_L^1^ (0.302 g, 1.02 mmol). The resultant mixture was stirred at room temperature for 20 min and a dark green solution was obtained. The solution was then filtered and the solvent was allowed to evaporate slowly at room temperature. After 2 d, single crystals suitable for X-ray diffraction were isolated, washed 3 times with cold methanol and dried over silica gel.

Yield: 0.523 g (68%, with respect to Cu). Anal. Calcd for C_14_H_14_Cu_3_N_10_O_16_: C, 21.87; H, 1.84; N, 18.22. Found: C, 21.83; H, 1.81; N, 18.17. IR (KBr; cm^−1^): 3448 ν(OH), 1610 ν(C=N), 1382 ν(NO_3_), 1252 ν(C–O)enolic and 1154 ν(N–N). UV–Vis λ_max_ (MeOH, nm (ε, LM^−1^ cm^−1^)): 752 (309), 820 (286), 390 (16,248), 282 (22,632), 254 (31,818). ESI-MS(+): *m/z* 770 [M+H]^+^ (100%).

### Synthesis of [Cu_2_(H_2_L^2^)(NO_3_)(H_2_O)_2_]_n_(NO_3_)_n_ (2)

To a 25 mL methanol solution of H_4_L^2^ (0.340 g, 1.00 mmol), a 20 mL methanol solution of Cu(NO_3_)_2_**·**2.5H_2_O (0.670 g, 3.0 mmol) was added and the reaction mixture was stirred for 20 min at room temperature. The resultant dark green solution was filtered and the filtrate was kept in open air. Dark green single crystals suitable for X-ray diffraction analysis were isolated after 2 days. Crystals were washed 3 times with cold ethanol and dried over silica gel.

Yield: 0.450 g (72%, with respect to Cu). Anal. Calcd for C_17_H_18_Cu_2_N_6_O_12_ (2): C, 32.65; H, 2.90; N, 13.44. Found: C, 32.60; H, 2.86; N, 13.39. IR (KBr; cm^−1^): 3432 ν(OH), 2874 ν(NH), 1607 ν(C=O), 1384 ν(NO3), and 1154 ν(N–N). UV–Vis λ_max_ (MeOH, nm (ε, LM−1 cm−1)): 756 (296), 359 (14,436), 344 (18,322), 326 (16,862). ESI-MS(+): m/z 581 [(M-NO_3_)+H_2_O]^+^ (100%).

### X-Ray Measurements

Crystals of **1** were unstable at room temperature and without solvent. A good quality single crystal of **1** in the mother liquor was mounted in a capillary tube, sealed and measured at the temperature of *ca*. 150 K. A crystal of **2** was immersed in cryo-oil, mounted in a Nylon loop and measured at the temperature of 296 K. Intensity data were collected using a Bruker AXS PHOTON 100 diffractometer with graphite monochromated Mo-Kα (λ 0.71073) radiation. Data were collected using omega scans of 0.5° per frame and full sphere of data were obtained. Cell parameters were retrieved using Bruker SMART (Bruker, 2012) software and refined using Bruker SAINT (Bruker, 2012) on all the observed reflections. Absorption corrections were applied using SADABS (Bruker, 2012). Structures were solved by direct methods by using SHELXS (Sheldrick, 2000) and refined with SHELXL 2018 (Sheldrick, 2015). Calculations were performed using WinGX version 2014.1 (Farrugia, 2012). All non-hydrogen atoms were refined anisotropically. The H-atoms bonded to carbons were included in the model at geometrically calculated positions and refined using a riding model. U_iso_(H) were defined as 1.2U_eq_ of the parent aromatic and methylene groups and 1.5U_eq_ of the parent methyl ones. The hydrogen atoms attached to O were located in the difference Fourier map and refined with their isotropic thermal parameter set at 1.5 times the average thermal parameter of the parent oxygen atom. There was disordered solvent in the structure of **2** that could not be modeled (232 electrons in a void of 471 Å^3^). Platon Squeeze (Spek, 2009) was used to remove that electron density. Least square refinements with anisotropic thermal motion parameters for all the non-hydrogen atoms and isotropic for the remaining atoms were employed. Crystal structures data are provided in the Supplementary Material.

### Typical Procedures and Product Analysis for Catalysis

The catalytic microwave-assisted (MW) peroxidative oxidation of 1-phenylethanol was undertaken in a focused Anton Paar Monowave 300 reactor equipped with a rotational system and an IR temperature detector. To a cylindrical pyrex tube (10 mL), 2.5 mmol alcohol, 5 μmol catalyst **1** or **2** (0.2 mol% *vs*. substrate) and 70% aqueous solution of *tert*-BuOOH (5 mmol) were added. The tube was sealed and placed in the microwave reactor under stirring and irradiation (5 or 20 W) at 80 or 120°C respectively, for 0.5 h. In the end, the reaction mixture was cooled to room temperature, 150 μL of benzaldehyde (internal standard) and 2.5 mL of MeCN (for substrate and organic products extraction) were added. The reaction mixture was stirred for 10 min and in the end a sample (1 μL) was taken from the organic phase and analyzed by GC using a FISONS gas chromatograph (GC 8000) with a FID detector and a capillary column (DB-WAX, column length: 30 m; internal diameter: 0.32 mm) (He as the carrier gas) and the Jasco-Borwin v.1.50 software. The samples were injected at 240°C. Initially the temperature was held at 120°C for 1 min, then elevated 10°C/min up to 200°C and maintained at this temperature for 1 min. Assignment of product peaks was done by comparison with chromatograms of pure commercial samples.

## Results and Discussion

### Syntheses and Characterization

The aroylhydrazone Schiff base *N'*^1^,*N'*^2^-bis(pyridin-2-ylmethylene)oxalohydrazide (H_2_L^1^) and *N'*^1^,*N'*^3^-bis(2-hydroxybenzylidene)malonohydrazide (H_4_L^2^) have been used to synthesize Cu(II) coordination polymers. Reactions of Cu(NO_3_)_2_·2.5H_2_O with those Schiff bases result in the formation of the 1D coordination polymers [Cu_3_(L^1^)(NO_3_)_4_(H_2_O)_2_]_n_ (**1**) and [Cu_2_(H_2_L^2^)(NO_3_)(H_2_O)_2_]_n_(NO_3_)_n_ (**2**). In **1** and **2** the coordination forms of the ligands are different (Scheme 1). Generally, in the presence of a base, aroylhydrazone coordinates to the metal center via the *iminol* (*enol*) form. The *amide* (*keto*) form is typically observed when reacting with a metal salt of the weak conjugated base of strong acid, e.g., nitrate, chloride or sulfate (Sutradhar et al., 2015a, 2016a). The treatment of H_2_L^1^ with Cu(NO_3_)_2_·2.5H_2_O yields the 1D polymer **1** with the *iminol* (*enol*) coordination form of the ligand without addition of a base. However, this is probably due to the presence of the basic pyridine moiety in H_2_L^1^ which helps to deprotonate the *enolic* hydrogen of another molecule during complex formation. In accord, the ligand exhibits the usual *amide* (*keto*) coordination in polymer **2**, where no such basic moiety is present in the ligand.

All the characteristic bands of the corresponding coordinated tridentate anionic ligand are found in the IR spectra of **1** and **2**, *viz*., 3,448, 1,610, 1,252, and 1,154 ν(N–N) cm^−1^ for **1** and 3,432, 2,874, 1,607, 1,384, and 1,154 cm^−1^ for **2**. In addition, the presence of nitrate was found at *ca*. 1,382 cm^−1^. In the UV-Vis spectra, both complexes **1** and **2** exhibit intense ligand to metal charge transfer transitions (LMCT) in the range of 254–390 nm (Figures S1,S2). In **1**, less intense absorption bands at 752 and 820 nm are due to d–d transitions attributable to ^2^B_1g_ → ^2^A_1g_ and ^2^B_1g_ → ^2^E_1g_ transitions, suggesting a distorted octahedral geometry (Figure S1) (Sutradhar et al., 2016b; Sureshbabu et al., 2019). In the case of **2** a less intense absorption band is observed at 756 nm (Figure S2) in accord with square pyramidal coordination geometries at the Cu(II) centers (Sutradhar et al., 2016b; Sureshbabu et al., 2019), which is in agreement with the structures obtained by single crystal X-ray analysis. The ESI-MS spectrum of compound **1**, in methanol solution (see experimental section), displays the molecular ion peak at *m/z* = 770 [**1**+H]^+^ (100%). For **2**, the peak at *m/z* 581 (100%) suggests the absence of the non-coordinated nitrate ion and the addition of one water molecule. The magnetic properties and catalytic activity toward solvent-free microwave-assisted oxidation of 1-phenylethanol of **1** and **2** are discussed in the following sections.

### Description of the Crystal Structures

**Figure 1:**
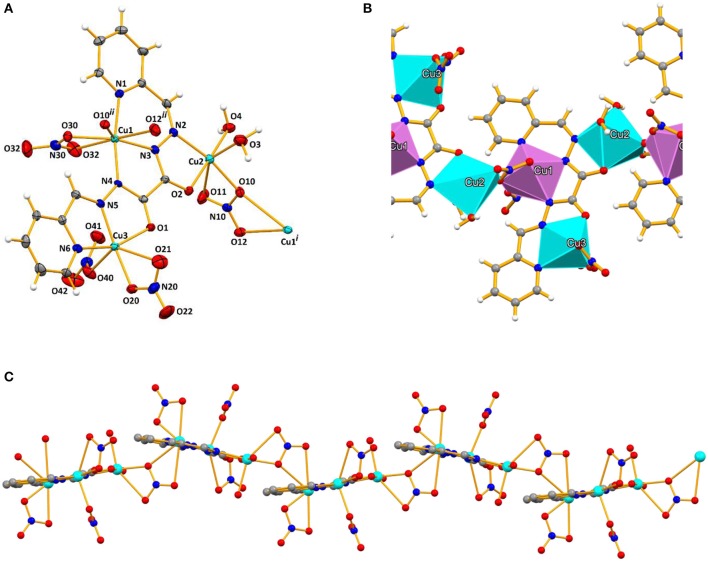
**(A)** Ellipsoid plot of **1** with partial atom numbering scheme, **(B)** a fragment of its 1D polymeric chain with the metals in polyhedral representations (coordination number six represented in blue, and seven in violet), and **(C)** a view of the smooth wave-like nature of the chain. Symmetry operation to generate equivalent atoms: (i) 1.5–x, –y,1/2+z; (ii) 1.5–x, –y, −1/2+z.

**Figure 2:**
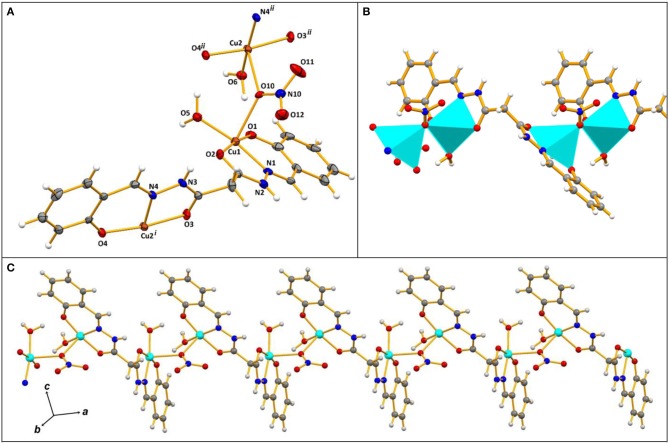
**(A)** Ellipsoid plot of **2** with partial atom numbering scheme; **(B)** a fragment of 1D polymeric chain with the metals in polyhedral representation; **(C)** a view of the 1D chain. The nitrate counter-ions are omitted for clarity. Symmetry operations to generate equivalent atoms: (i) 1+*x, y, z*; (ii) −1+*x, y, z*.

Single crystals suitable for X-ray analysis were isolated upon slow evaporation of a methanolic solution of **1** or **2** at room temperature. Crystals of **1** are unstable at room temperature and loose crystallinity in the absence of the mother liquor. A special precaution was taken (mentioned in experimental section) to analyze the structure. Crystallographic data for **1** and **2** are summarized in Table S1 and selected dimensions (bond angle and lengths) are presented in Table S2. Figures 1, 2 represent the structures of the respective complexes.

The multidentate ligand (L^1^)^2−^ in **1** is planar and chelates to three Cu(II) ions through the *NNN*, the *NNO* and the *NO* pockets affording one 6-membered and four 5-membered metallacycles (Figure 1A). The copper cations are slightly displaced (maximum of 0.197 Å) toward the same side of the least-square plane of the organic ligand. Despite the planarity of (L^1^)^2−^, its derived 1D coordination polymer has a smoth wave-like shape imposed by the coordination mode of one of the nitrate anions behaving as bridging 1κ*OO*':2κ*OO*” donors to Cu1 and Cu2; the least-square planes of adjacent (L^1^)^2−^ ligands make angles of 19.84°. The remaining nitrate ligands work as bidentate (to Cu1 and to Cu3) and as monodentate (to Cu3) ligands. The two water molecules are coordinated to the same copper cation (Cu2). The metal ions assume coordination numbers six (Cu2 and Cu3) and seven (Cu1), with the coordination polyhedron of Cu3 (Figure 1B) having no contact atom with the other polyhedra; those of Cu1 and Cu2 share an O_nitrate_ atom. The *intra*chain Cu…Cu distances through the azine bridges are of 4.736 and 4.794 Å, and of 4.451 Å through the nitrate bridge. The minimum *inter*chain Cu…Cu distance is of 5.692 Å.

The asymmetric unit of **2** contains two Cu(II) ions, one (H_2_L^2^)^2−^, one nitrate and two water ligands, and a nitrate counter-ion. The organic ligand in **2** behaves as a hexadentate chelator acting as an *NOO* donor for each of the Cu(II) ions, therefore giving rise to two six- and two five-membered metallacycles (Figure 1A). Bridging monodentate nitrate anions connect the {Cu_2_(H_2_L^2^)}^2−^ moieties and generate a 1D polymeric chain running along the crystallographic *a* axis (Figure 1C). The copper cations adopt square pyramidal geometries (τ_5_ = 0.04 for Cu1 and 0.05 for Cu2) and share the O_nitrate_ atom that stands in the axial position (Figures 2A,C). In view of the binding mode of the nitrates, the shortest Cu…Cu distance of 4.028 Å is along such ligands, while that along the hydrazone group exclusively (6.469 Å) is much longer than in **1** (see above). Resulting from the presence of a central methylene group in (H_2_L^2^)^2−^ the ligand is highly twisted at this level, with the least-square planes of the two phenyl(methylene)acetohydrazide moieties making angles of 84.85°.

### Magnetic Properties

**Figure 3:**
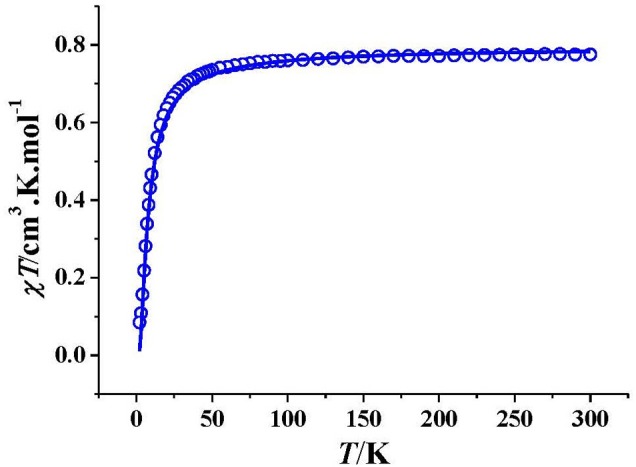
Plot of χ*T vs*. *T* for **2**. The solid line represents the best theoretical fitting.

The dc magnetic susceptibility of **2** was measured under 2000 Oe field (Figure 3). The room temperature χ*T* value of 0.775 cm^3^ K mol^−1^ is a little larger than 0.750 cm^3^ K mol^−1^ calculated for two isolated Cu^2+^ ions. The χ*T* value for **2** decreases gently with decreasing temperature in the range of 300–25 K, and then decreases sharply. The magnetic data at 50–300 K follow the Curie-Weiss law, with *C* = 0.785 cm^3^.K.mol^−1^ and Θ = −3.20 K (Figure S3). The small negative Θ value suggests existence of weak antiferromagnetic interaction in **2**.

**Figure 4:**
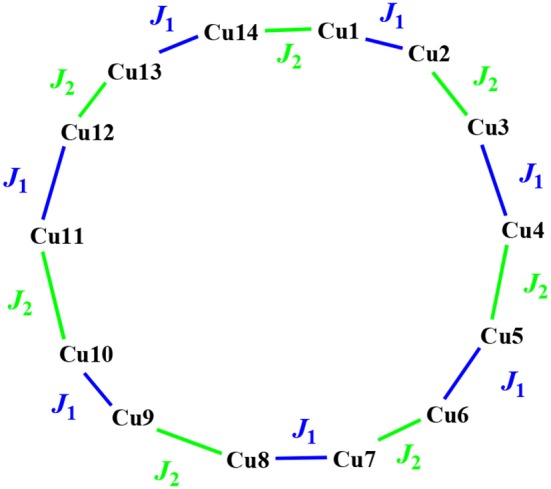
Magnetic topology of Cu_14_ cycle in **2** (the copper numbers do not show crystallographically independent copper ions, but rather are used to distinguish them).

Based on the crystal structure, complex **2** is an alternative copper(II) chain compound, so two *J*_1_ and *J*_2_ parameters are necessary to describe the magnetic interaction, which are mediated through the ligand bridge and the O_nitrate_ bridge, respectively. The magnetic data could be fitted by MagPack software using a Cu_14_ cluster loop approximation (Figure 4). The Hamiltonian of the Cu_14_ cluster loop with alternative *J*_1_ and *J*_2_ magnetic coupling constants is as follows: *H* = −2*J*_1_(*S*_Cu1_*S*_Cu2_+*S*_Cu3_*S*_Cu4_+*S*_Cu5_*S*_Cu6_+*S*_Cu7_*S*_Cu8_+*S*_Cu9_*S*_Cu10_ + *S*_Cu11_*S*_Cu12_+*S*_Cu13_*S*_Cu14_)−2*J*_2_(*S*_Cu2_*S*_Cu3_+*S*_Cu4_*S*_Cu5_+*S*_Cu6_*S*_Cu7_ + *S*_Cu8_*S*_Cu9_+*S*_Cu10_*S*_Cu11_+*S*_Cu12_*S*_Cu13_+*S*_Cu14_*S*_Cu1_). The best fitting gave *J*_1_ = −1.84 cm^−1^, *J*_2_ = −4.35 cm^−1^, *g* = 2.10 and *R* = 1.6 × 10^−4^. Both *J*_1_ and *J*_2_ values are small, suggesting that the antiferromagnetic interaction in **2** is weak. The *J*_2_ value is more negative than that of *J*_1_, indicating that the antiferromagnetic interaction through the ligand bridge is weaker than that through the O_nitrate_ bridge.

### Catalytic Studies Toward Microwave (MW) Assisted Oxidation of a Secondary Alcohol Under Solvent-Free Conditions

**Scheme 2:**
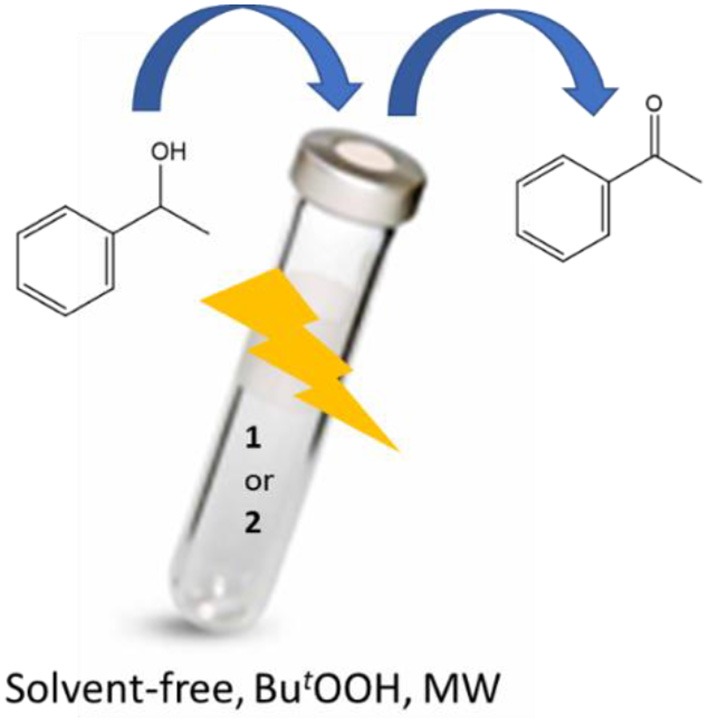
MW-assisted solvent-free oxidation of 1-phenylethanol to acetophenone.

Cu(II) complexes **1** and **2** have been investigated as catalysts for the homogeneous oxidation of a secondary alcohol, 1-phenylethanol, to the corresponding ketone using *tert*-butylhydroperoxide (*tert*-BuOOH, TBHP, aq. 70%, 2 eq.) as oxidizing agent. The oxidation reactions were performed typically at 80 or 120°C, low power (5 or 20 W) microwave irradiation (MW), 0.5 h reaction time without additional solvent (Scheme 2). Results are summarized in Table 1.

**Table 1:** Solvent-free MW-assisted oxidation of 1-phenyethanol using **1** and **2** as catalysts precursors (selected data)^a^.

**Entry**	**Substrate**	**Temperature (°C)**	**Additive (μmol)**	**Yield^b^ (%)**	**TON [TOF (h^**−1**^)]^c^**
**1**					
1	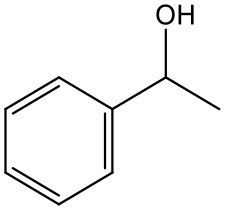	80	-	15	150 (300)
2		120	-	68	334 (668)
3		80	TEMPO (30)	40	202 (404)
4		120	TEMPO (30)	>99	588 (1.2 × 10^3^)
5		80	TFA (50)	22	111 (222)
6		80	TFA (100)	25	124 (248)
7		80	Hpca (50)	13	102 (204)
**2**					
8	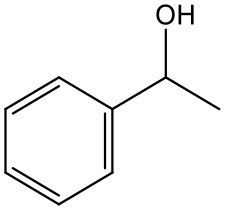	80	-	11	94 (188)
9		120	-	47	244 (488)
10		80	TEMPO (30)	33	163 (326)
11		120	TEMPO (30)	>99	522 (1.0 × 10^3^)
12		80	TFA (50)	16	81 (162)
13		80	TFA (100)	20	99 (198)
14		80	Hpca (50)	6	32 (64)

a *Reaction conditions: 2.5 mmol of substrate, 5 μmol (0.2 mol% vs. substrate) catalyst **1** or **2**, 5 mmol of t-BuOOH (70% aq. solution), 0.5 h, 80 or 120°C, MW irradiation (5 or 20 W, respectively)*.

b *Molar yield (%) based on substrate, i.e., moles of product per 100 mol of substrate, determined by GC*.

c *Turnover number = number of moles of product per mol of metal catalyst; TOF = TON per hour (values in brackets)*.

Complexes **1** and **2** proved to be essential for the formation of acetophenone, since under similar reaction conditions and in the absence of these metal catalysts only traces of the desired product were detected. For catalytic tests performed only with the free ligand, no product was detected.

Under the studied conditions, the assays with copper catalysts **1** and **2** have shown comparable yields. For instance, for both **1** and **2** and using TBHP as oxidant at 120 °C, in the presence of TEMPO (2,2,6,6-tetramethylpiperydil-1-oxyl) radical, the almost complete conversion of 1-phenylethanol to the desired product is achieved after the short period of 30 min with a TON of 588 and 522, respectively (Table 1, entries 4 and 11, respectively).

**Figure 5:**
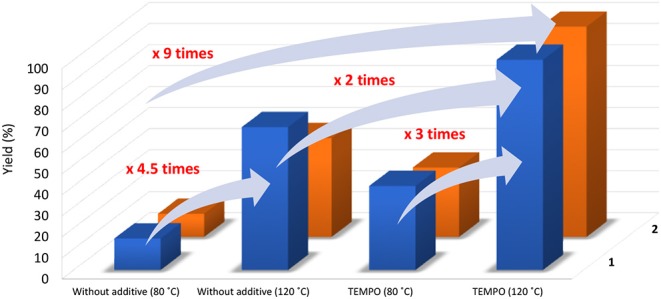
Influence of temperature and of presence of TEMPO additive on the yield of acetophenone from oxidation of 1-phenylethanol catalyzed by **1** and **2**. Reaction conditions: 2.5 mmol of substrate, 5 μmol (0.2 mol% *vs*. substrate) catalyst **1** or **2**, 5 mmol of *t*-BuOOH, 0.5 h, 80 or 120°C (5 or 20 W, respectively).

For these encouraging results, the effect of temperature and the presence of TEMPO, a chemically stable nitroxyl radical which has emerged as a promoter for the metal catalyzed transformation of alcohols to the corresponding carbonyl products (Gamez et al., 2004; Sheldon and Arends, 2004; Figiel et al., 2007, 2009; Sheldon, 2008; Ahmad et al., 2009), seem to be determinant. Reactions carried out under the same reaction conditions but at 80°C and in the absence of any additive did not go beyond 15 and 11% in the presence of **1** and **2**, respectively (Table 1, entries 1 and 8, respectively) whereas when carried out at 120°C it was possible to observe a significant increase in yield to 68 and 47% for **1** and **2**, respectively (Table 1, entries 2 and 9, respectively). The combination of these two parameters, temperature at 120°C and presence of TEMPO radical (Figure 5), resulted in the complete conversion of 1-phenylethanol into acetophenone and TOFs up to 1200 h^−1^ (Table 1, entries 4 and 11, for **1** and **2**, respectively).

**Figure 6:**
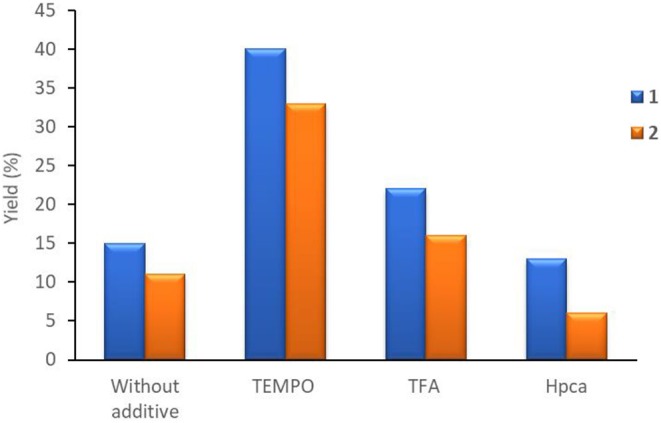
Influence of different additives on the yield of acetophenone from oxidation of 1-phenylethanol catalyzed by **1** and **2**. Reaction conditions: 2.5 mmol of substrate, 5 μmol (0.2 mol% vs. substrate) catalyst **1** or **2**, 5 mmol of *t*-BuOOH, 0.5 h, 80°C (5 W MW irradiation).

Considering the promoting effect of acid co-catalysts observed for other Cu-catalyzed oxidation systems (Sutradhar et al., 2016a,c, 2018), which are believed to accelerate the oxidation reactions by improving the oxidation properties of the complexes and by creating unsaturated metal centers (Sutradhar et al., 2015c), the influence of acidic additives on the acetophenone product yield was explored. The addition of trifluoroacetic acid (TFA) has a slight beneficial effect on both catalytic systems, resulting, for example, into a maximum yield of 25% (Table 1, entry 6), in the presence of **1** (at 80°C) (Figure 6). In contrary, the addition of the heteroaromatic 2-pyrazynecarboxylic acid (Hpca) has the opposite effect (Figure 6), i.e., a yield drop is observed when 50 μmol was used [*n*(acid)/*n*(catalyst **1** or **2**) = 10] (Table 1, entries 7 and 14, for **1** and **2**, respectively).

The promoting effect of TEMPO suggests the involvement of a radical mechanism which possibly involves the formation of *t*-BuOO and *t*-BuO radicals by a metal-assisted oxidation or reduction of *t*-BuOOH by a Cu^II^ or a Cu^I^ center, respectively (Gephart et al., 2012; Dronova et al., 2014), the latter behaving as an H-atom abstractor from the alcohol (Rothenberg et al., 1998; Mahdavi and Mardani, 2012; Frija et al., 2016; Sutradhar et al., 2016a,b, 2018; Ma et al., 2019).

## Conclusions

In this study we have successfully synthesized two 1D Cu(II) coordination polymers using two different multidenate aroylhyrazone Schiff bases. The X-ray crystallographic study indicates that the two ligands show different coordination behaviors. The *N'*^1^,*N'*^2^-bis(pyridin-2-ylmethylene)oxalohydrazide (H_2_L^1^), having a basic pyridine moiety, coordinates in the *iminol* form, whereas the *amide* form is observed in the case of **2**. Both the ligand bridge and the nitrate bridge in **2** mediate weak antiferromagnetic interaction. The effects of temperature and of the aditive TEMPO dramatically increase the catalytic efficiency of both Cu(II) compounds in the microwave-assisted oxidation of 1-phenylethanol to acetophenone under solvent-free conditions.

Our study concerns an attempt to design Cu(II) coordination polymers with interesting magnetic properties and also for use as catalyst toward the development of environmentally friendly alcohol oxidation catalytic systems.

## Data Availability Statement

All datasets generated for this study are included in the article/Supplementary Material. The crystallographic datasets generated for this study can also be found in the Cambridge Crystallographic Data Center (https://www.ccdc.cam.ac.uk/structures/) under the identifiers 1945551 and 1945552.

## Author Contributions

MS: overall planning, synthesis and characterization of catalysts, and manuscript writing. EA: catalytic studies and manuscript writing. TB: catalytic studies, GC analysis and manuscript writing. MG: single crystal X-ray diffraction analysis. C-ML: Magnetic studies and manuscript writing. AP: manuscript reading and correcting.

### Conflict of Interest

The authors declare that the research was conducted in the absence of any commercial or financial relationships that could be construed as a potential conflict of interest.

## Supplementary Material

Data sheet 1 Crystal structures data.
