# Supplementary material for: 1D Copper(II)-Aroylhydrazone Coordination Polymers: Magnetic Properties and Microwave Assisted Oxidation of a Secondary Alcohol
Source: Front Chem. 2020 Mar 6;8:157. doi: 10.3389/fchem.2020.00157 (PMC7069101; doi:10.3389/fchem.2020.00157)
Supplement: Supplementary file 1 [file Table_1.docx]

**Electronic Supplementary Information**

Aroylhydrazone-Copper(II) 1D Polymers: Magnetic Properties and Microwave Assisted Oxidation of Secondary Alcohol

Manas Sutradhar^1,^*, Elisabete C.B.A. Alegria^1,2^, Tannistha Roy Barman^1^, M. Fátima C. Guedes da Silva^1^, Cai-Ming Liu^3^, and Armando J. L. Pombeiro^1,^*

**Table S1.** Crystal data and structure refinement details for **1** and **2**.

|  | **1** | **2** |
| --- | --- | --- |
| Empirical formula | C_14_H_14_Cu_3_N_10_O_16_ | C_17_H_18_Cu_2_N_6_O_12_ |
| Formula Weight | 768.97 | 625.45 |
| Crystal system | Orthorhombic | Triclinic |
| Space group | *P*2_1_2_1_2_1_ | *P*¯1 |
| Temperature/K | 296(2) | 148(2) |
| *a*/Å | 11.0540(3) | 9.780(4) |
| *b*/Å | 13.7402(6) | 12.550(5) |
| *c*/Å | 16.2327(6) | 12.687(3) |
| α/° | 90 | 80.193(7) |
| *β/*° | 90 | 87.957(5) |
| γ/° | 90 | 81.029(5) |
| *V* (Å^3^) | 2465.49(16) | 1515.6(10) |
| *Z* | 4 | 2 |
| D_calc_ (g cm^–3^) | 2.072 | 1.371 |
| *μ*(Mo K*α*) (mm^–1^) | 2.665 | 1.461 |
| Rfls. collected/unique/observed | 33336/4357/3606 | 16979/5192/3377 |
| *R*_int_ | 0.0715 | 0.0727 |
| Final *R*1 *^a^*, *wR*2 *^b^* (*I* ≥ 2*σ*) | 0.0338, 0.0598 | 0.0819, 0.2374 |
| Goodness-of-fit on *F*^2^ | 1.044 | 1.019 |

*^a^* R *=* Σ||*F_o_*|–|*F_c_*||/Σ|*F_o_*|; *^b^* wR(F^2^) = [Σw(|F_o_|^2^ – |F_c_|^2^)^2^/Σw|F_o_|^4^]^½^

**Table S2.** Selected bond distances (Å) and angles (º) in complexes **1** and **2**.

**1**

| O1—Cu3 | 2.018(4) | O40—Cu3 | 2.231(5) |
| --- | --- | --- | --- |
| O2—Cu2 | 1.955(4) | N1—Cu1 | 1.987(4) |
| O3—Cu2 | 2.313(6) | N2—Cu2 | 2.009(5) |
| O4—Cu2 | 1.938(4) | N4—Cu1 | 1.983(4) |
| O10—Cu2 | 1.995(4) | N5—Cu3 | 1.927(5) |
| O20—Cu3 | 1.958(4) | N6—Cu3 | 2.026(5) |
| O30—Cu1 | 2.003(4) |  |  |
| N3—Cu1—N4 | 80.48(18) | O2—Cu2—O3 | 93.2(2) |
| N3—Cu1—N1 | 91.65(19) | O10—Cu2—O3 | 96.55(19) |
| N4—Cu1—N1 | 170.0(2) | N2—Cu2—O3 | 89.2(2) |
| N3—Cu1—O30 | 158.0(2) | N5—Cu3—O20 | 172.3(2) |
| N4—Cu1—O30 | 94.76(18) | N5—Cu3—O1 | 79.66(18) |
| N1—Cu1—O30 | 94.80(19) | O20—Cu3—O1 | 100.51(17) |
| O4—Cu2—O2 | 177.7(2) | N5—Cu3—N6 | 80.22(19) |
| O4—Cu2—O10 | 91.02(17) | O20—Cu3—N6 | 98.2(2) |
| O2—Cu2—O10 | 89.00(16) | O1—Cu3—N6 | 157.94(16) |
| O4—Cu2—N2 | 98.22(18) | N5—Cu3—O40 | 105.4(2) |
| O2—Cu2—N2 | 81.54(17) | O20—Cu3—O40 | 82.2(2) |
| O10—Cu2—N2 | 169.22(17) | O1—Cu3—O40 | 100.55(18) |
| O4—Cu2—O3 | 89.2(2) | N6—Cu3—O40 | 93.4(2) |

**2**

| O1—Cu1 | 1.903(5) | O6—Cu2 | 1.961(6) |
| --- | --- | --- | --- |
| O2—Cu1 | 1.980(5) | O10—Cu1 | 2.308(6) |
| O3—Cu2^i^ | 1.980(5) | O10—Cu2 | 2.340 (6) |
| O4—Cu2 | 1.902(5) | N4—Cu2i | 1.933(7) |
| O5—Cu1 | 1.948(6) | N1—Cu1 | 1.937(7) |
| O1—Cu1—N1 | 92.9(3) | O4—Cu2—N4^ii^ | 93.0(3) |
| O1—Cu1—O5 | 93.4(3) | O4—Cu2—O6 | 93.6(2) |
| N1—Cu1—O5 | 170.0(2) | N4^ii^—Cu2—O6 | 169.4(2) |
| O1—Cu1—O2 | 172.5(3) | O4—Cu2—O3^ii^ | 172.6(3) |
| N1—Cu1—O2 | 81.6(2) | N4^ii^—Cu2—O3^ii^ | 80.9(2) |
| O5—Cu1—O2 | 91.4(2) | O6—Cu2—O3^ii^ | 91.7(2) |
| O1—Cu1—O10 | 85.8(2) | O4—Cu2—O10 | 85.7(2) |
| N1—Cu1—O10 | 101.3(2) | N4^ii^—Cu2—O10 | 100.2(2) |
| O5—Cu1—O10 | 87.0(2) | O6—Cu2—O10 | 88.5(2) |
| O2—Cu1—O10 | 100.3(2) | O3^ii^—Cu2—O10 | 99.6(2) |

Symmetry codes: (i) *x*+1, *y*, *z*; (ii) *x*-1, *y*, *z*.


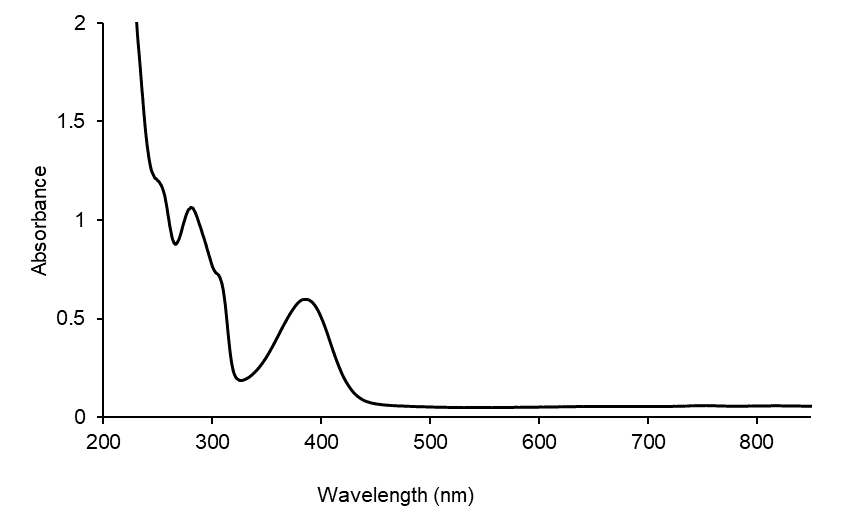

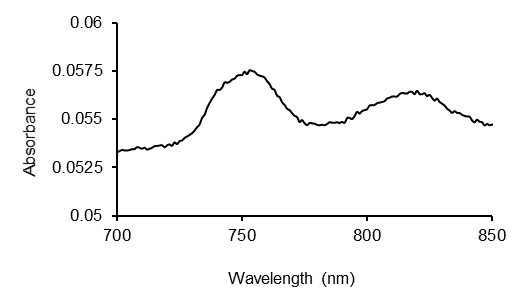


**Figure S1.** UV-Vis spectrum of **1** in methanol.


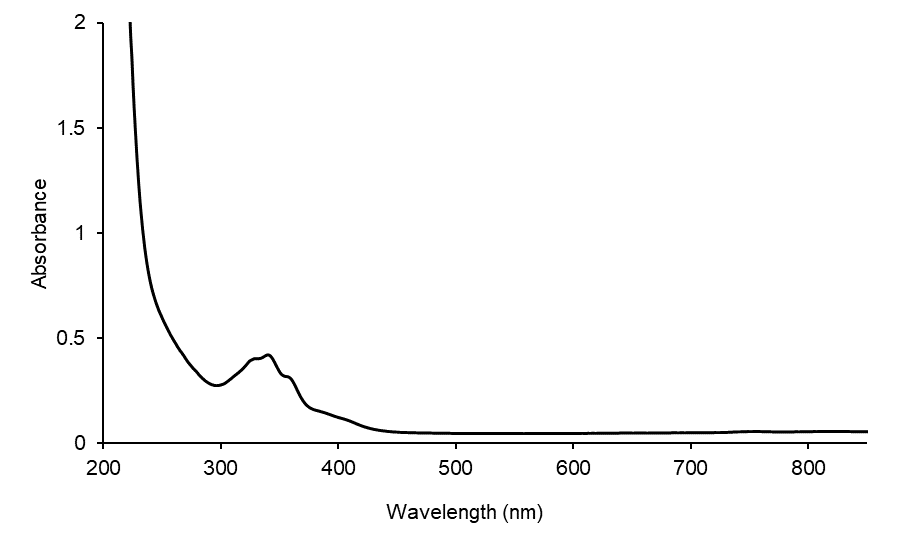

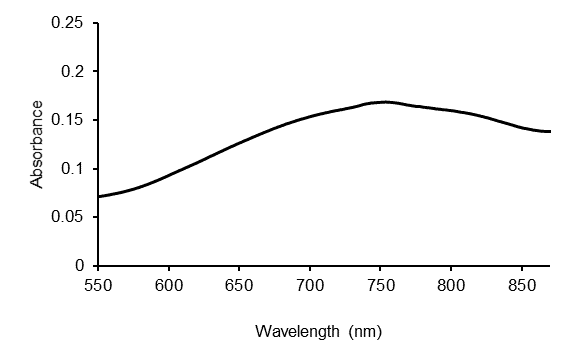


**Figure S2.** UV-Vis spectrum of **2** in methanol.

**Figure S3.** Plot of *χ^-1^* vs *T* for **2**.
